# Security Enhancement of Wireless Sensor Networks Using Signal Intervals

**DOI:** 10.3390/s17040752

**Published:** 2017-04-02

**Authors:** Jaegeun Moon, Im Y. Jung, Jaesoo Yoo

**Affiliations:** 1 School of Electronics Engineering, Kyungpook National University, 80 Daehakro Buk-gu, Daegu 702701, Korea; jaekun34@knu.ac.kr; 2 School of Information and Communication Engineering, Chungbuk National University, 1 Chungdaero Seowon-gu, Cheongju 28644, Korea; yjs@chungbuk.ac.kr

**Keywords:** IoT, wireless sensor network, WPAN, Bluetooth, simple secure pairing, signal interval

## Abstract

Various wireless technologies, such as RF, Bluetooth, and Zigbee, have been applied to sensor communications. However, the applications of Bluetooth-based wireless sensor networks (WSN) have a security issue. In one pairing process during Bluetooth communication, which is known as simple secure pairing (SSP), the devices are required to specify I/O capability or user interference to prevent man-in-the-middle (MITM) attacks. This study proposes an enhanced SSP in which a nonce to be transferred is converted to a corresponding signal interval. The quantization level, which is used to interpret physical signal intervals, is renewed at every connection by the transferred nonce and applied to the next nonce exchange so that the same signal intervals can represent different numbers. Even if attackers eavesdrop on the signals, they cannot understand what is being transferred because they cannot determine the quantization level. Furthermore, the proposed model does not require exchanging passkeys as data, and the devices are secure in the case of using a fixed PIN. Subsequently, the new quantization level is calculated automatically whenever the same devices attempt to connect with each other. Therefore, the pairing process can be protected from MITM attacks and be convenient for users.

## 1. Introduction

Many sensors are in wide use today as components of different devices (temperature sensors in home or car heating systems, smoke alarms, etc.). Otherwise, these sensors are standalone devices connected to a network, typically used to monitor industrial processes, equipment, or installations [1]. Various wireless technologies, such as RF, Bluetooth, and Zigbee, have been applied to sensor communications. Wireless senor networks (WSN) are being expanded, connected, and integrated to other networks. Therefore, they are a type of Internet of Things (IoT). In addition, electronic devices that use wireless personal area networks (WPANs) are increasing rapidly with the advent of the IoT era [2]. However, the threat posed by individuals attempting to eavesdrop on WPAN information or WSN and use them inappropriately is real [3]. To address this problem, many WPANs or WSNs have authentication processes [4,5,6,7].

One well-known WPAN or WSN is Bluetooth, which creates a link key for devices to authenticate each other in their communication and extract an encryption key through this pairing process [8]. In addition, Bluetooth has protocols for processing the pairing. This process is composed of a sequence of giving and receiving information and comparing the values transferred. With the Bluetooth 2.1+ BR/EDR version, simple secure pairing (SSP) has been defined and used to process pairing. Although the current Bluetooth version is 4.2, Bluetooth continues to use SSP to support compatibility with previous versions [9].

**Figure 1:**
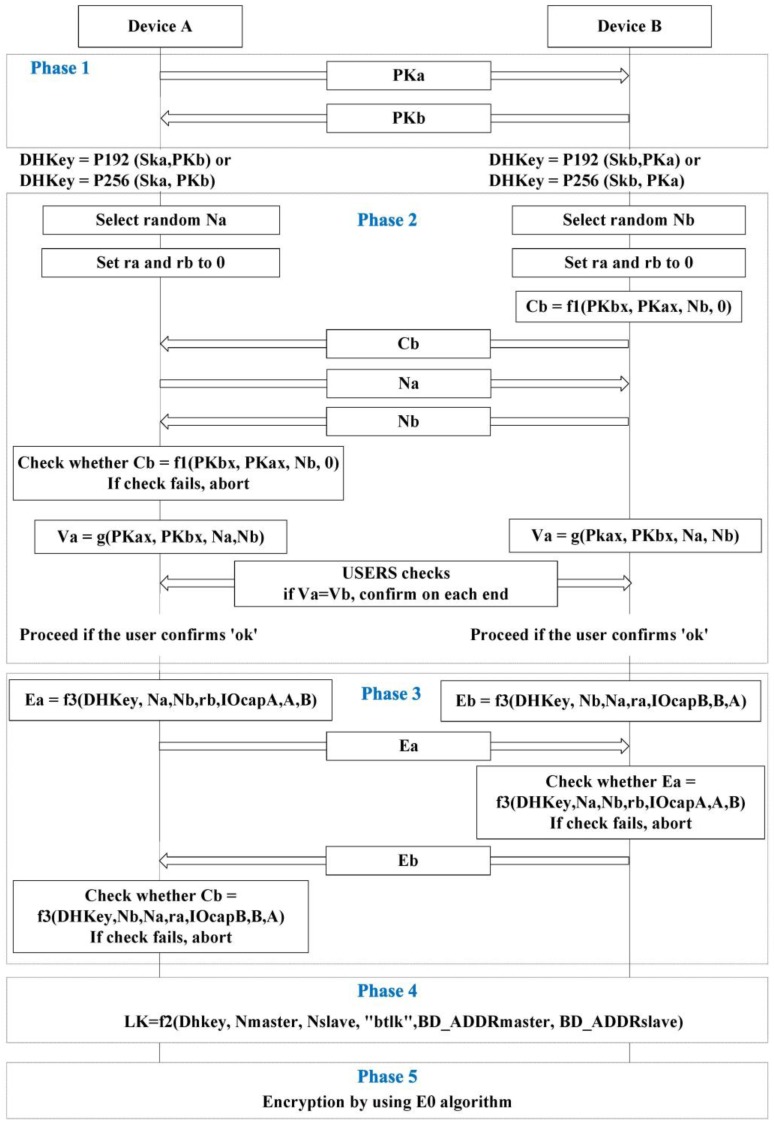
Simple secure pairing with a numeric comparison association model [9].

As shown in Figure 1 and Table 1, SSP consists of five phases. Phase 1 uses the Diffie-Hellman (DH) key exchange. However, DH key exchange has vulnerabilities to man-in-the-middle (MITM) attacks [10].

To resolve the MITM attack problem, SSP adopts an association model: either numeric comparison, passkey entry, Just Works, or out of band (OOB). One of these association models adopted in Phase 2 differs based on the I/O capability of the electronic devices that connect to one another. The I/O capability of the devices to be connected is exchanged before Phase 1 of SSP. The devices then choose a single association model in Phase 2. Phases 3 and 4 check and combine both types of information. Phase 4 then creates a link key used for authentication. An encryption key is created and encryption is conducted in Phase 5. However, this study does not describe the detailed process that follows Phase 2 because the proposed model employs a strain of passkey entry and numeric comparison in Phase 2. Table 2 describes the association models based on the I/O capability of the connected devices. DisplayOnly refers to those devices having only a display. DisplayYesNo is for those devices having a display and input channel for inputting “Yes” or “No.” KeyboardOnly refers to those devices having only a keyboard. NoInputNoOutput is for those devices having no I/O capability. KeyboardDisplay refers to those devices having both a display and keyboard.

OOB is known to be resistant to MITM attacks. However, the electronic devices must support other communication methods or channels in order to apply OOB.

In numeric comparison, the devices display a number and users confirm whether the number displayed corresponds to the device to be connected in the pairing process [9]. The association model for numeric comparison can prevent MITM attacks. However, a display and confirmation process is required for every connection. Moreover, if the same passkey is used, this association model cannot prevent MITM attacks [11]. Furthermore, if the devices do not have displays, numeric comparison cannot be applied [9]. However, the proposed model does not require display equipment.

During the passkey entry process, a given user inputs the passkey into a device and the devices to be paired compare both passkeys [9]. For the passkey entry, each connection requires user input. If the same passkey is used for every connection, this association model cannot prevent MITM attacks [11].

Just Works is nearly identical to numeric comparison. However, Just Works does not display a number. Therefore, the devices to be connected omit the comparison process conducted by users and proceed to the subsequent step. Without displaying a number, the devices cannot be alerted to MITM attacks [12,13,14,15].

In this study, an enhanced SSP that uses signal intervals is proposed to improve SSP security and user convenience. Specifically, the proposed scheme improves Phase 2 of SSP with numeric comparison.

This SSP includes the challenge-respond method [9]. When a random number or nonce is transferred, the signal interval that expresses the nonce is delivered. Using a pre-defined function and the nonce transferred previously, the matching rule based on the quantization level between the signal interval and nonce is renewed. Specifically, the matching rule defines the quantization level with which the signal intervals match nonces. Due to delay, error, or other interference from physical signals, it is difficult for the intended signal interval to be delivered precisely. To reduce the error sensitivity of the physical signals, this study introduces the quantization level of the signal interval instead of using the analog characteristics of the physical signal [16]. Since the quantization level is renewed, the device that knows the previous nonce can determine the next new signal interval.

During the initial step, the devices to be initially connected do not have any previous shared nonces. Therefore, users must input a 64-bit passkey. However, this input is required only once, that is, when the two devices are connected for the first time. Since the passkey entered by the user is not transferred as data, should a third party eavesdrop on the signals, it cannot determine the passkey information.

Therefore, the proposed model can also be applied in the case of a fixed PIN. This fixed PIN is saved in the device that does not have any input method/input device. Since the devices can adopt the fixed PIN, this association model can be applied to a case in which one of two devices does not have input capability if they have the same fixed PIN. Regular users of Bluetooth generally use Bluetooth communication to connect laptops, desktops, or smartphones to speakers, headsets, or earphones.

As indicated in Table 2, current devices use Just Works because laptops, desktops, and smartphones employ displays and keyboards. On the other hand, speakers, headsets, and earphones are NoInputNoOutput. However, if the proposed model is adopted, the devices can connect with each other using a fixed PIN. Moreover, more than two devices attempting to pair can recognize each other and prevent MITM attacks. In addition, the devices do not need to support other communication methods or channels.

The proposed method has an advantage in that third parties attempting to disguise themselves cannot determine the nonce shared between the devices to be connected. This is because they cannot calculate the quantization level renewed at every connection. Unlike current SSPs, the proposed method can minimize user interference. Since the passkey entered by a user is not transferred as data, if a third party eavesdrops on the signals, it cannot determine the passkey information. Additionally, since information to be transferred is expressed by the time interval of the physical signals, the attacker that cannot determine the quantization level cannot determine the meaning of the signal intervals. Due to the threat of leaking the quantization level when a fixed quantization level is used, the transferred number is used to renew the existing quantization level. Therefore, MITM attacks can be prevented.

This study considers related works in Section 2. Section 3 describes the proposed scheme of the new association model. Section 4 describes the results of experiments conducted in various environments and analyzes the security of the proposed Scheme. Security of proposed model is analyzed in Section 5. Section 6 summarizes and concludes the study.

## 2. Related Works

Electronic devices exchange certain information in order to authenticate one another or create a key. In anonymous communication, devices basically exchange a given value and then authenticate each other. In ZigBee, authentication is performed using the devices’ pre-shared master key, ID, and nonce. By compounding the master key, ID, and nonce, a link key is created [17]. The master key is either transferred or pre-installed. In wireless LAN, access control that uses a service set identifier (SSID), address authentication through media access control (MAC), shared key authentication, or the IEEE802.1x method, is used [18,19,20,21]. These methods are also implemented during communication among devices.

However, because such communication is vulnerable to sniffing, some countermeasures are required [3]. For example, transferring the master key through communication is vulnerable to replay attacks [22]. Therefore, many studies have been conducted on key distribution [23,24] to resolve these kinds of threats. Since Bluetooth has the same vulnerabilities, SSP is adopted. However, SSP is not secure. Therefore, many methods have been proposed to supplement or replace SSP [25,26,27]. One method exists in which information is stored using the device ID in a database [25]. This method uses a pre-shared password and extracts a key from the password. The key encrypted by a public key is then delivered. However, this database must be pre-shared. Moreover, if the ID of the new connecting device does not exist in the database, the device cannot connect. This is another case of giving and receiving information through communication. Therefore, the sniffing threat remains. If the inner algorithm is revealed through sniffing, this method is not secure.

Another proposal assumes a situation in which many master devices are present in a piconet, and two given devices among the master devices create a key [26]. The key is then delivered to other devices by means of multicast and used for authentication. However, this method cannot be used for two-device communication and cannot check for an attacker attempting to infiltrate the piconet.

In addition, distinguishing a node or device is possible by using the analog character of the signals generated by the node [28]. SSP is processed using this character [27]. However, this method employs an OOB channel to prevent MITM attacks. Therefore, a device that does not support this type of channel for other communication cannot employ this method. Moreover, the recognition rate is only 70%. Thus, identical devices that attempt to authenticate each other may fail authentication at a rate of 30% [28]. In addition, this method requires an additional server to verify authentication results.

By contrast, the proposed method does not require a piconet or an additional server. In addition, the accuracy of our method was approximately 95% in our experiment.

Another suggested use of quantization is for an electronic device to project data onto the space containing a random array. This would be performed after the device extracts biological data. This model then applies dynamic thresholding and encrypts the data before transferring them [29]. Furthermore, another proposed error recovery method exists that outperforms the existing dithered quantization method because it uses random quantization of the source signal [29,30].

## 3. Enhanced SSP Using Signal Intervals

The scheme proposed shows that the time interval between consecutive physical signals can also be used to improve SSP. As indicated in [11], passkey entry has security flaws. Therefore, this study proposes an enhanced SSP using signal intervals. This SSP is based on numeric comparison and passkey entry for pairing devices that possess no display window. In addition, the proposed method does not require an input passkey when the passkey is saved to the devices. In this case, the counterpart device must have its input subpart device to acquire the passkey. Therefore, the other pairing device does not require its own input subpart device, such as a keyboard [12,13,14,15]; the legacy passkey entry requires an input passkey at each pairing. By contrast, the proposed scheme does not require an input passkey except at the initial connection. After the initial connection, the current nonce is used instead of the passkey.

### 3.1. Assumption

It is assumed that the received signal strength indication (RSSI) of the signals is higher than −70 dbm to prevent packet loss.

In addition, because attackers can falsify device addresses, each device uses its resolvable private random device address [9], which is composed of 48 bits that, themselves, are composed of 24 bits each of random value and a hash of the random value. The devices require a local or peer identity resolving key (IRK) to create the hash value. To share IRK, the devices should connect at least once. IRK is shared during the first pairing. Specifically, IRK is shared following the pairing process to protect it from sniffing. In Section 3.6, it is assumed that the devices have already shared IRK. A resolvable private random device address can support device identification by the hash value, even when the device address has changed.

In addition, the pairing devices share necessary information such as I/O capability and Bluetooth address. The proposed scheme must share additional information. This study proposes that the information be shared during the paging process.

Finally, RTC is necessary to obtain date information. The date information is used to calculate the quantization level. The RTCs of the two devices to be paired can be synchronized using a method proposed by [31] during paging process; IP or GPS can be used to synchronization of RTCs. If the devices fail to synchronization, the mixture of IRK of two devices can be used instead of date information.

### 3.2. Random Number Sharing

**Figure 2:**
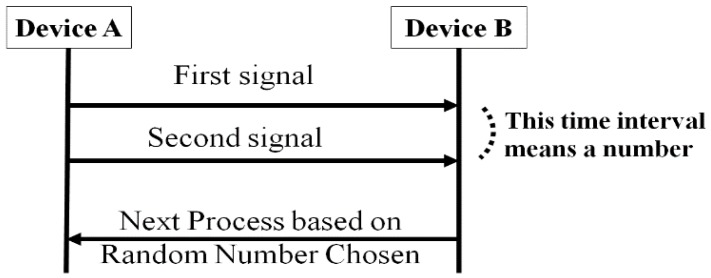
Random number selection using signal interval.

Figure 2 illustrates the nonce sharing process that uses the time interval between two consecutive signals. The information delivered by the two signals is the maximum signal interval between consecutive signals. These sets of data should be as short as possible to save energy. Device B calculates the values necessary to process SSP with the transmitted nonce.

**Figure 3:**
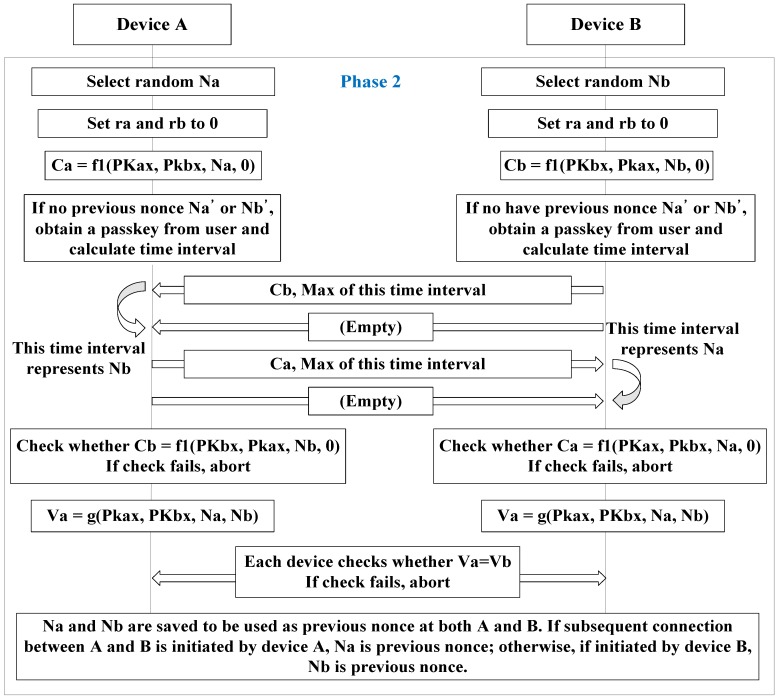
SSP Phase 2 that adopts the proposed scheme.

Figure 3 shows the enhanced version of Phase 2 of the SSP. The reason both parties must check Ca and Cb is to create a pre-existing nonce for both devices. Both ra and rb are set to 0 in order to adopt the legacy scheme of numeric comparison. Ca and Cb are used to check ra and rb are properly transmitted. In addition, Va and Vb are exchanged to identify each other.

Due to communication delay, signal loss, or an abnormal device status, the intended time interval might not be transferred. Therefore, quantization is used to reduce error sensitivity caused by physical environments. Therefore, this scheme can obtain tolerance, like the conversion process of an analog signal to a digital signal.

**Figure 4:**
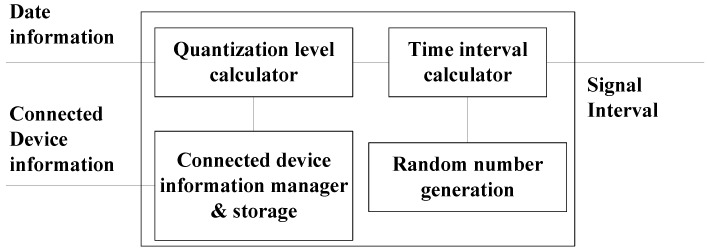
Architecture of the device paired that generates the signals representing random numbers.

Figure 4 is a block diagram showing the chip that calculates the quantization level and the signal interval that generates the signals. The date information shows the time in ms units after 1 January 1970. The connected device information shows the Bluetooth device address. The previously-saved nonce is searched for using this address. If no nonce was previously saved, the user must input the passkey. The quantization level is calculated using the pre-existing nonce or passkey input, as well as the date information, which are all shared by the two devices. Since the signal interval is calculated by multiplying the quantization level and random number, the signal receiver can determine the sender’s random number by dividing the signal interval by the quantization level.

### 3.3. Quantization Level Management

#### 3.3.1. Quantization Level Initialization

Information for devices previously connected and the pre-existing nonce used are saved and managed as shown in Figure 4. During the initial connection, because the used storage does not contain any information about the devices to be connected, the user must input a 64-bit passkey. Otherwise, a fixed passkey can be used based on the devices’ I/O capabilities. Since a passkey is used, the quantization level is different at each pairing. However, pairing devices can achieve the same quantization level if the same passkey is input. This input is required whenever new devices attempt to connect. However, once the passkey has been entered for the device pair, subsequent connections between the device pair do not require a new passkey for each connection.

**Figure 5:**
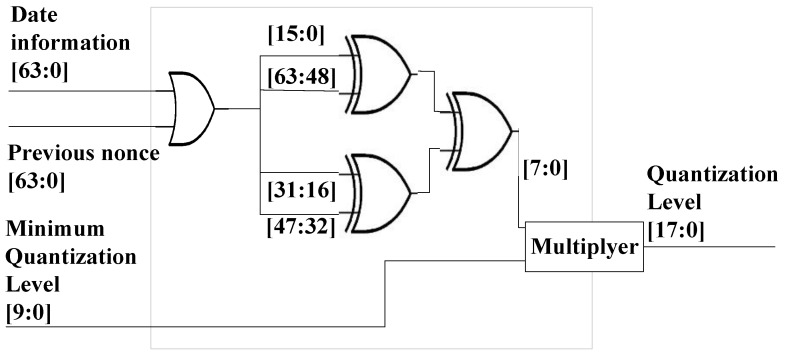
Example of quantization level calculator.

The quantization level is calculated at the subsequent circuit as shown in Figure 5. In this circuit, the date information is in ms units after 1 January 1970. For example, if the date information is 1445242302710 and the passkey is 0, the value is 1445242302710 after the OR gate is passed. Subsequently, a value of 129 is extracted from the lower eight bits after passing the XOR gate. The minimum quantization level is 625 µs in Bluetooth [9]. Therefore, the result of the quantization level is 80.625 ms. After the quantization level is calculated, as shown in Figure 5, the devices multiply the nonce provided by the random number generator by the quantization level. In this example, if the time interval unit is ms, a small nonce can result in a large time interval. However, if the quantization level unit is μs, the time interval can be reduced. When the time interval is delivered to the receiver, the nonce represents the quotient of the division of the time interval by the quantization level.

Since Bluetooth can synchronize time with circumjacent devices [9], the devices can use the same time. If a connection is successful, information on the connecting device and random number used is saved to create the next new quantization level.

Figure 5 provides an example of a quantization level calculator that conducts the renewal process. The quantization level calculator can be modified based on the practical specifications of the real devices.

#### 3.3.2. Quantization Level Renewal

If no renewal occurs, an adversary can obtain information about a quantization level when many pairing cases are observed. Therefore, the quantization level must be renewed.

When devices to be connected have, in fact, already connected (at an earlier time), the data recorded from that earlier connection exists in storage, as indicated in Figure 4. Figure 4 shows the architecture of the device to be paired in order to renew the quantization level. The new quantization level is calculated using the previous connection information. Two random variables exist. The previous nonce is a random number created by the device that initiates the connection. In Figure 3, the random numbers are Na and Nb. The previous nonce is Na if the subsequent connection is initiated by device A. Since the saved nonce is the same for the devices to be connected, each device can calculate the same result using this shared nonce.

**Figure 6:**
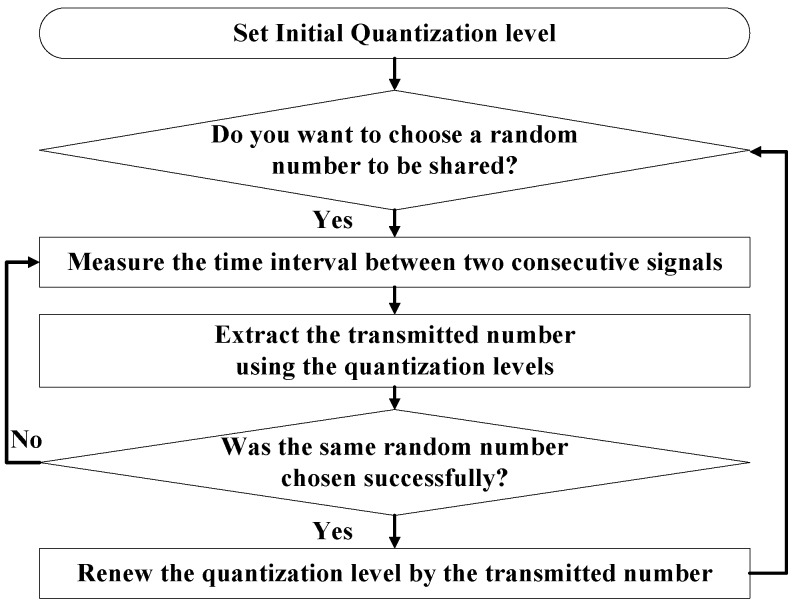
Quantization-level renewal process.

Figure 6 shows the algorithm for the renewal process. Through this algorithm, the devices can avoid leaking the renewed quantization level whenever the devices establish new connections. When a new connection is established, the nonce changes. Therefore, the quantization level is renewed whenever a new connection is established. The same signal intervals can represent different numbers as the quantization level changes. Therefore, the devices can protect their data because the third party cannot determine the real value shared.

### 3.4. Countermeasures for Packet Loss

The loss rate in wireless communication is greater than that in wired communication.

As explained in Section 3.3.1, when devices acquire 80.625 ms of quantization level, if the random number generator changes the nonce to 8, the signal interval becomes 645 ms. However, one of two consecutive signals can be lost. To solve this problem, this study proposes that every signal have its data express the maximum signal interval as shown in Figure 3. The receiver waits for the next signal during the maximum signal interval. If the maximum signal interval expires, the receiver will conclude that the second signal is lost, and require a new signal interval. If an empty signal is delivered first, the receiver will know that the first signal is lost. The maximum signal interval is calculated using Equation (1):

M = (1 + X) P(1)

where M and P are the maximum and intended signal intervals, respectively. X should be a value between 0 and 1. However, if X is very small, the signal can be ignored when a slight delay occurs. For example, if P is 645 ms and X is 0, M is 645 ms. If the signal delay is 1 ms, a 646-ms signal interval would be sensed. However, this value would be invalid. In this case, the receiver requires that the sender retransmit two consecutive signals. In addition, if X is a large value, the receiver must wait for the lost packet during the surplus time. Therefore, it is proper for X = 0.5, academically speaking. Nonetheless, the value of X can be adjusted to reduce the error rate caused by the poor status of the physical communication. The transmitter sets X to 0.5 initially and can adjust X based on the authentication success rate.

### 3.5. Transmitted Value Management

Since attackers can mimic the authentication of normal devices or attempt to use brute force during attacks using clues from the maximum signal interval, when a counterpart transmits the wrong authentication value, the devices stops the pairing process, as in the current association model.

### 3.6. Counterfeit Detection

With the proposed model, the nonce can be recognized by estimating the signal interval. Thus, if any attacker watches the signals and copies them, the accurate value cannot be transferred to the receiver because the signal interval between two consecutive signals is different from the intended interval. In other words, the receiver cannot be authenticated. To prevent this situation, when a device perceives an imitated signal, the device creates a new device address. If old and new device addresses from identical devices exist, the receiver ignores the old device addresses.

## 4. Evaluation

To analyze the security of the proposed scheme, the attack scenario suggested by [11], in which the passkey entry is violated, is used here. This is because the proposed method in this study uses passkey entry and numeric comparison to improve the security of the current passkey entry.

**Figure 7:**
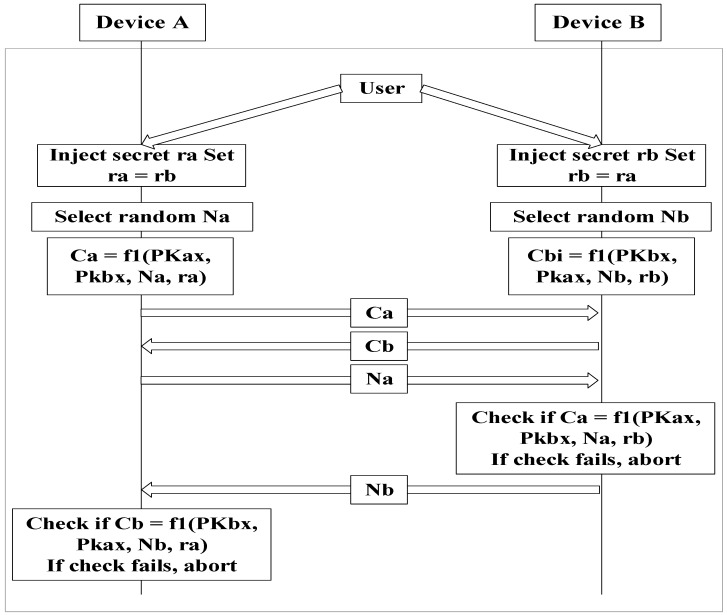
Passkey entry [9].

The passkey entry method is illustrated in Figure 7. The process is repeated using a bit number of the passkey. Note that ra and rb in each round are the values of the bits of the passkey. The passkey entry uses ra, rb, Na, and Nb of the last round in Phase 3 as well.

The point of these attack scenarios is to implement to identify an MITM in Phase 1 and determine the last bit of the passkey. All information used after Phase 1 can be monitored because it is transmitted in plain text. The last bit of the passkey is then used for ra and rb. In this case, ra and rb are identical because the passkeys of the pairing devices are the same. The attacker can easily determine rx by calculating Cx and then comparing the calculated and transmitted values using legitimate pairing devices because the last bit is 0 or 1.

Moreover, the legacy SSP has a security flaw a fixed passkey is used. This is because ra and rb are always the same in all pairing processes (they are either 0 or 1). In addition, Nx is transmitted in plain text. Therefore, an adversary can obtain all information during the SSP process. By contrast, an adversary cannot determine Na and Nb easily using the time interval of our scheme because this interval is already included in the nonce (Nx) and uses all bits of the passkey. This proposed method does not directly reveal both the passkey and nonce.

Therefore, the proposed scheme can be applied when a pairing device has a fixed passkey. A fixed passkey is not renewed. However, after the first pairing, the pre-existing nonce is used instead of date information. Therefore, an adversary cannot assume or detect the quantization level and thus extract the nonce from the current time interval.

In addition, Table 3 shows the comparison of existing protocols with our approach. The proposed method does not need any extra device, but shows high accuracy.

## 5. Experiments

### 5.1. Accuracy Test Settings

An experiment was conducted in which a nonce used to create a key or to authenticate devices is represented by a signal interval between two consecutive signals of Bluetooth low energy (BLE). BLE was used because Bluetooth classic has its time slot of 625 µs and the advertisement function of BLE has the signal interval that is a time slot of 625 µs in the range from 20 ms to 10.24 s. Some delay is added to the signal interval [9]. The delay of BLE can be negated by adjusting the quantization level.

The checkpoint is the location to which an intended value is transferred. It is assumed that the initial quantization level had been determined in advance and that other information used to authenticate each device had been exchanged, with the exception of the nonce. After nonce transmission, a device does not respond to the other device and simply displays that value. In addition, this test ignores the manner in which authentication fails, i.e., the renewal of the quantization level through the authentication value, and saving or deleting the transferred value.

Bluetooth beacon signals were used in this experiment. A desktop equipped with a Bluetooth dongle and using Ubuntu OS transmitted the beacon signals to a laptop equipped with the same Bluetooth dongle and Ubuntu OS. Specifications for the desktop PC were as follows: Intel core i7-4790 CPU and 8 GB RAM. The laptop had an Intel core i5-3230M CPU and 4 GB RAM.

The transmitted nonce was compared with the number calculated by the laptop by estimating the signal interval between two consecutive signals. The Bluetooth dongle was a ZIO BT40 of Bluetooth CSR 4.0 made by ZIO company in goyang of Korea, which supports Bluetooth 4.0. The distance between two devices was 50 cm. The intended signal intervals were 62.5 ms, 313 ms, and 625 ms. The valid value was assumed to be between the intended time intervals of −10 ms and +10 ms. Each signal contained information that the maximum signal interval was 100 ms, 500 ms, and 1 s. For the beacon, the feedback for packet loss was set so that it need not be considered. In addition, the device was set so that it waited for subsequent signals that were repeatedly transmitted. If the device was not a beacon, the feedback might not have been required when packet loss occurred. Through such feedback, the transmitter could generate new signals. The given signal intervals represent values such as 100, 500, 1000.

### 5.2. Quantization Level Management

#### 5.2.1. Effect of Maximum Signal Interval

Before conducting an actual experiment, a test was performed to determine the influence of the maximum signal interval.

The distance between two devices was 50 cm. In addition, 62.5 ms of the time interval represents a value of 100. The signal interval was ignored if it exceeded the maximum, and was measured again. In this situation, the average signal intervals, standard deviation, and accuracy were estimated.

Table 4 lists the results. By adapting the maximum signal interval, an improved outcome was obtained with higher accuracy and a time interval that was close to that intended in comparison to the outcomes obtained when not adapting it.

Table 5 describes the accuracy of the transferred nonce through the time interval between signals. The results indicate an accuracy of approximately 95% to 98%. This result is considered in order to determine the quantization level. In addition, the time for the quantization level should be longer than the overhead of the estimated time interval in order to reduce the error rate.

#### 5.2.2. Signal Interval between Correct Values Based on Distance

**Figure 8:**
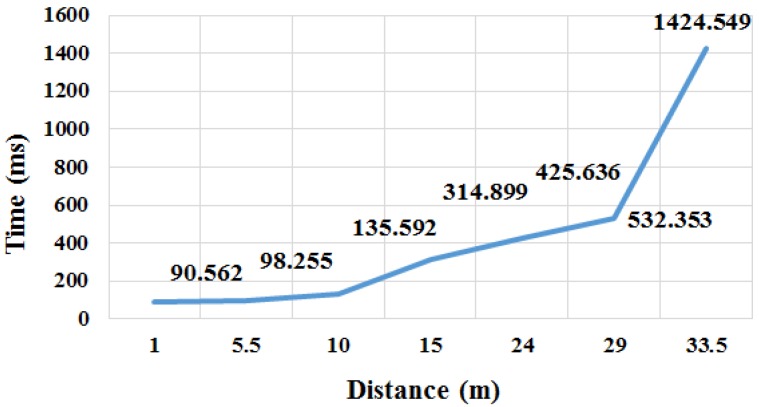
Random number sharing affected by signal loss.

Figure 8 shows the time necessary to obtain an accurate nonce based on the distance between the devices that are to be connected. In other words, Figure 8 shows the time interval between accurate nonce acquisition and the next accurate nonce acquisition. The experiment showed how the proposed model was affected by signal loss, meaning that if the communication environment was bad, the scheme does not work effectively. No obstacle was present between the two devices. The time interval required to obtain accurate nonces increased rapidly at a distance longer than 10 m. In other words, the proposed model is effective within 10 m.

#### 5.2.3. Interference Effect

Wireless communication can be affected by interference. An experiment was conducted to show the interference effect. The number of devices accessing BLE signal in the same channel was set as a variable. The BLE signal interval was chosen as 20 ms, which is the minimum BLE signal interval [9], to show an obvious inference effect in this experiment (except BLE delay).

**Figure 9:**
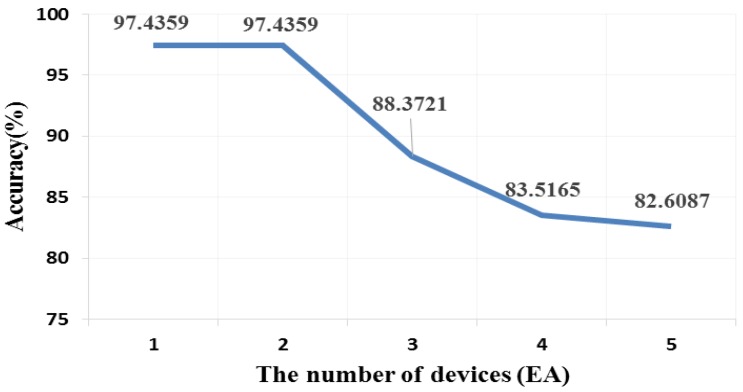
Random number sharing affected by interference effect.

Figure 9 describes the interference effect depending on the number of devices sending signals. This experiment was designed to show an environment of the proposed scheme in a busy channel. The number of devices means the number of devices sending or receiving a packet every 20 ms. The factor of the distance causes packet loss. Therefore, the accuracy would be similar in any distance when the minimum signal interval is set. Conversely, the interference effect may lead to some delay and then decrease accuracy. When all five devices sending signals every 20 ms were on different channels from the device transferring nonce, the result was similar to that shown in Table 4.

This experiment shows an extreme case because the BLE interval in this experiment was set to the minimum. In an ordinary case, the beacon signal interval is set to be longer to reduce power consumption. Further, when a channel is busy, the device can change the advertisement channel to diminish the interference effect [9]. If all channels are busy, the quantization level can be greater for better performance.

#### 5.2.4. Implementation of Random Number Mapping

Many studies have obtained sensitive information meant to be processed through a secure route within hardware chips [32,33,34]. To improve security, hardware implementation has often been considered [35]. To this effect, we implemented a field-programmable gate array (FPGA), namely, the Spartan6 XC6LX from Xilinx, and analyzed it using ISE based on Figure 6. The date information contained the current time expressed by a 64-bit integer, and the quantization level result was expressed by 8-bit data.

Table 6 lists the time required to obtain a result after the input of each signal. As indicated in Table 6, the devices could acquire high-speed processing if a digital circuit was produced to implement the proposed model. In this experiment, the device required a processing time of within 16 ns.

### 5.3. Overhead Analysis

In the proposed method, a busy waiting time occurs for receiving two signals. However, this waiting time is not long when considering the communication time of Bluetooth signals.

The time intervals in the program used in our experiment were set to 62.5, 313, and 625 ms. However, the estimated values had about 4~5 ms of errors on average because of advertisement delay [9]. Thus, this error is reduced.

To consider the storage space required, this study used the circuit shown in Figure 6. In this circuit, the nonce and Bluetooth device address size were set to 8 and 6 bytes, respectively. Therefore, the required storage was 14 bytes per device. This means that if the device saved information about 1000 devices to be connected, it required 1.4 Kbytes. If the devices used 16 bytes of nonce, the devices required 22 bytes per device.

## 6. Conclusions

This study proposed an improved SSP to transfer nonces through signal intervals between two consecutive signals. A non-encrypted nonce was transferred because the devices had not already created a key in SSP. Therefore, attackers could eavesdrop on, and collect information related to, key creation. In this association model, even if attackers eavesdropped on the signals, because they could not determine the quantization level, they were unable to determine what was being transferred. Furthermore, we showed that the proposed model does not require exchanging passkeys as data and that devices are secure when using a fixed PIN. The devices to be connected obtained the shared passkey that was inputted initially by the user or that was fixed in order to calculate the quantization level. Subsequently, the new quantization level was calculated automatically whenever the same devices attempted to connect with each other.

In addition, we showed that applying the proposed scheme to other WSN is possible when the protocol includes a nonce transmission process. In other words, the nonce can be delivered using a time interval.

Although numeric comparison and passkey entry require user confirmation whenever devices connect, we showed that the proposed model does not require additional user interference, except in the initial device connection. In WPAN, the pairing devices are constant. Therefore, the proposed association model can provide convenience and is effective for users.

In addition, we suggested a hardware implementation to prevent software hacking. However, measuring the interval between two consecutive signals may be occasionally incorrect in multitasking environments. Moreover, this method is inefficient in high packet loss rate environments.

Therefore, applying the proposed random number sharing to near-field communication of IoTs would be effective. The target IoT devices to be connected should possess low specifications that cannot or do not require multitasking or overload applications.

## Figures and Tables

**Table 1:** Term of simple secure pairing with numeric comparison from Figure 1.

Expression	Description
PKx	Public key of device x
PKxx	x-coordinate of PKx
SKx	Secret (private) key of device x
DHKey	Diffie-Hellman key
Nx	Nonce (unique random value) from device x
rx	Random number generated by device x
Cx	Commitment value from device x
f1()	Used to generate the 128-bit commitment values Ca and Cb (ex. f1(PKbx,PKax,Nb,0) = $HMAC-SHA-256_{Nb}(PKb||PKa||0^{8})\mathrm{mod}2^{128}$)
f2()	Used to compute the link key (ex. f2(PKax,PKbx,Na,Nb) = $SHA-256(PKax||PKbx||Na||Nb)\mathrm{mod}2^{32}$)
f3()	Used to compute the check values Ea and Eb in Authentication Stage 2 (ex. f3(DHkey,Na,Nb,rb,IOcapA,A,B) = $HMAC-SHA-256_{DHkey}(Na||Nb||rb||IOcapA||A||B)$)
g()	Used to compute numeric check values
IOcapx	IO capabilities of device x
x	Bluetooth device address of device x
Ex	Check value from device x
LK	Link key
Vx	Confirmation value on device x

**Table 2:** Association model based on I/O capability [9].

Initiator	Responder	Association Model
DisplayOnly	KeyboardOnly	Passkey Entry
	KeyboardDisplay	
DisplayYesNo	DisplayYesNo	Numeric Comparison
	KeyboardDisplay	
	KeyboardOnly	Passkey Entry
KeyboardOnly	Any equipment except	Passkey Entry
	NoInputNoOutput	
KeyboardDisplay	DisplayOnly	Passkey Entry
	KeyboardOnly	
	DisplayYesNo	Numeric Comparison
	KeyboardDisplay	

**Table 3:** Comparison of existing protocols with our approach.

	Existing SSP [9]	Diallo [25]	Biswas [26]	Pasanen [27]	Our Study
MITM	△ (All scheme except numeric comparison)	△ (Connection of new device)	△ (Infiltrating attacker in the piconet)	X	X
Number of devices	2	3	At least 3	2	2
Accuracy	Not mentioned	Not mentioned	Not mentioned	About 70%	About 95%

O: Possible, △: Partially Possible, X: Impossible, (): Possible Case or Method.

**Table 4:** Effect of maximum signal interval.

Classification	Time Interval (ms)	Accuracy (%)
Adapted	67.15 ± 2.14	98.67
Not adapted	78.13 ± 28.2	81.58

**Table 5:** Random number mapping.

Random Number	Time Interval (ms)	Accuracy (%)
100	67.16 ± 2.92	98.67
500	317.23 ± 3.32	94.67
1000	629.81 ± 3.04	98.67

**Table 6:** Process time of random number mapping.

Input	Processing Time (ns)
Date information	12.202–15.675
Previous nonce	12.713–15.601
Minimum quantization level	10.882–12.714

## Abbreviations

WSN	Wireless Sensor Network
WPANs	Wireless Personal Area Networks
MITM	Man-In-The-Middle
SSP	Simple Secure Pairing
IoT	Internet of Things
OOB	Out of Band
DH	Diffie-Hellman
WPANs	Wireless Personal Area Networks
RSSI	Received Signal Strength Indication
IRK	Identity Resolving Key
FPGA	Field-Programmable Gate Array
